# Nanofluidic logic with mechano–ionic memristive switches

**DOI:** 10.1038/s41928-024-01137-9

**Published:** 2024-03-19

**Authors:** Theo Emmerich, Yunfei Teng, Nathan Ronceray, Edoardo Lopriore, Riccardo Chiesa, Andrey Chernev, Vasily Artemov, Massimiliano Di Ventra, Andras Kis, Aleksandra Radenovic

**Affiliations:** 1 Laboratory of Nanoscale Biology, Institute of Bioengineering, Ecole Polytechnique Federale de Lausanne (EPFL), Lausanne, Switzerland; 2 NCCR Bio-Inspired Materials, Ecole Polytechnique Federale de Lausanne (EPFL), Lausanne, Switzerland; 3 Laboratory of Nanoscale Electronics and Structures, Institute of Electrical and Microengineering & Institute of Materials Science and Engineering, Ecole Polytechnique Federale de Lausanne (EPFL), Lausanne, Switzerland; 4 Department of Physics, University of California, San Diego, La Jolla, CA USA

**Keywords:** Nanopores, Fluidics

## Abstract

Neuromorphic systems are typically based on nanoscale electronic devices, but nature relies on ions for energy-efficient information processing. Nanofluidic memristive devices could thus potentially be used to construct electrolytic computers that mimic the brain down to its basic principles of operation. Here we report a nanofluidic device that is designed for circuit-scale in-memory processing. The device, which is fabricated using a scalable process, combines single-digit nanometric confinement and large entrance asymmetry and operates on the second timescale with a conductance ratio in the range of 9 to 60. In operando optical microscopy shows that the memory capabilities are due to the reversible formation of liquid blisters that modulate the conductance of the device. We use these mechano–ionic memristive switches to assemble logic circuits composed of two interactive devices and an ohmic resistor.

## Abstract (web summary)

Nanofluidic devices with a large entrance asymmetry can function as memristive switches—operating on the second timescale and with a conductance ratio of up to sixty—and can be assembled into basic logic circuits.

## Main

A fundamental difference between artificial computers and biological brains is the nature of their information carriers. Computers rely on electrons and holes, while brains employ a range of different ions to process data^1^, using nanoscale synaptic ion channels to perform information processing and storage at energy costs orders of magnitude lower than solid-state digital circuits^2,3^. Understanding and harnessing ionic transport under nanometric confinement is a goal of nanofluidics^4^. This has been achieved in different geometries, including zero-dimensional nanopores^5,6^, one-dimensional nanotubes^7–9^ and two-dimensional (2D) slits^10–13^. Such platforms have been used to emulate biological ionic transport^8,9,14–25^ and could potentially be used to perform computational operations. Recent work has shown, in particular, that nanofluidic channels filled with aqueous electrolytes can store information by exhibiting a memristive effect^2,26–31^.

A memristive device, which can also be called a memristor^32^, is a passive two-terminal electrical component with a programmable conductivity that depends on its previous history of operation. Memristors can act as the artificial equivalent of biological synapses due to their ability to store information as a conductance value^33^, thus enabling data storage and processing with a single device. They can be used in electronic neural networks to adjust the connection strengths at nodes between crossbars, acting as the basic unit for brain-inspired, or neuromorphic, computing^34^.

Unlike previous fluidic memristors^35–37^, micropipettes coated with polyimidazolium brushes and bidimensional slits can exhibit memory effects in simple electrolytes within the electrochemical window of water, specifically at voltages below 1.23 V (refs. ^26,27^). For both devices, this behaviour is understood using phenomenological modelling by considering the combined effects of asymmetric entrances and slow interfacial diffusion, resulting in cycles of accumulation and depletion of ions inside the channel. Such a phenomenology recalls the induction of long-term potentiation through local changes in calcium ion concentration occurring in biological synapses^38,39^. Yet, direct experimental confirmations regarding the mechanisms of nanoscale fluidic memristors are still lacking, which restricts efforts to improve the performances of these devices.

The large volume occupied by micropipettes and the complex fabrication procedure of 2D slits, as well as the slow speed or small hysteresis displayed by these devices, also hinders their use as synaptic components on the circuit scale. To enable practical computing applications—such as logic operations, pattern recognition and image processing—it is essential to connect multiple memristive devices^28,40–44^. In this Article, we report devices—termed ‘highly asymmetric channels’ (HACs)—that are designed for circuit-scale neuromorphic nanofluidics. By combining electrokinetic measurements with optical observations, we show that the memory effect in the HACs arises from a combined mechano–ionic effect. With the HACs, we implement a nanofluidic logic operation by connecting two fluidic cells.

## Highly asymmetric channels as nanofluidic memristors

**Fig. 1:**
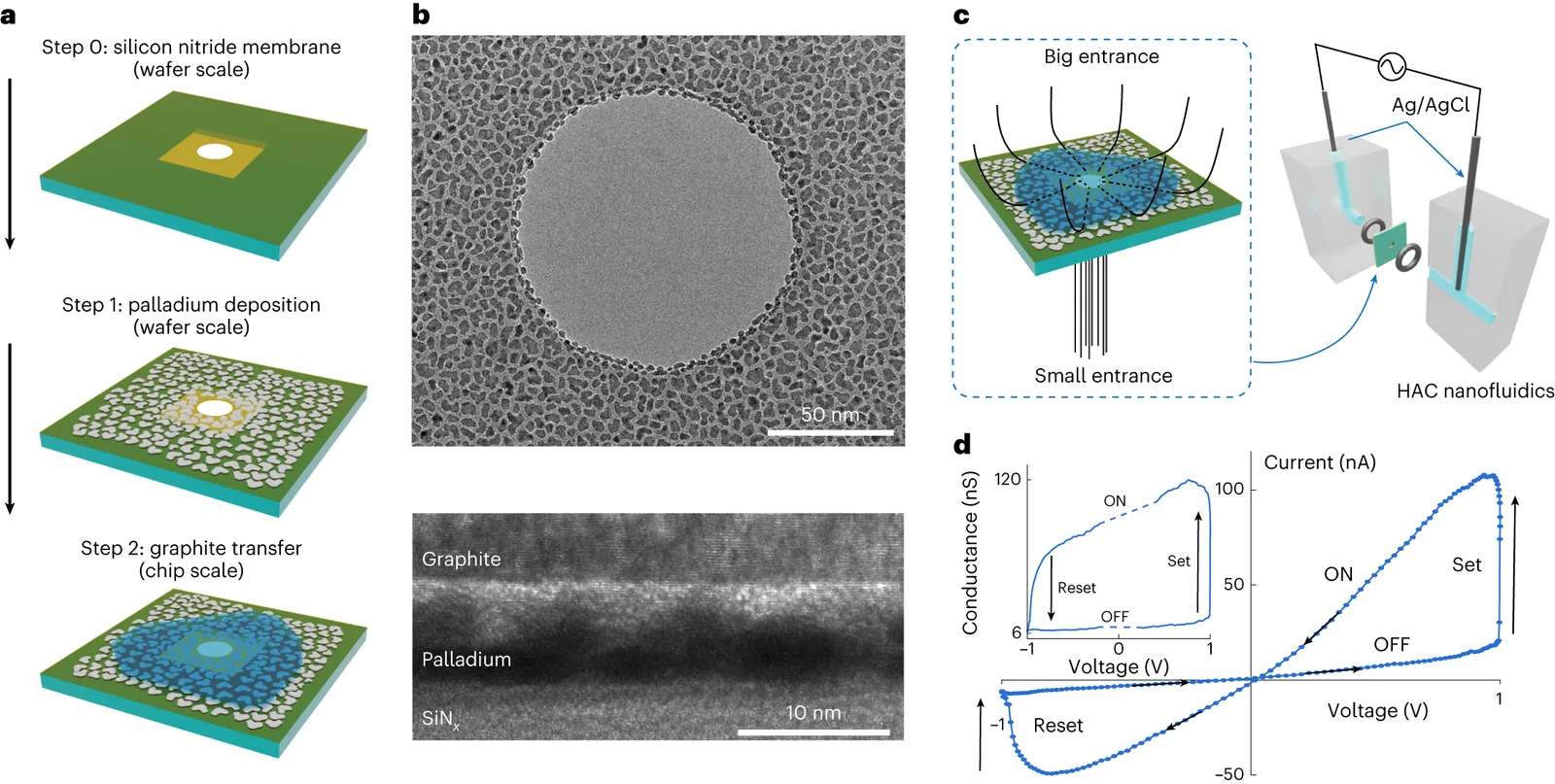
Highly asymmetric channels. **a**, Nanofabrication process flow. Step 0**:** the starting point is silicon chips (in green) with an SiN membrane (in yellow) of around 20 × 20 microns, a thickness of 20 nm and an aperture with a diameter of approximately 100 nm. Step 1**:** palladium islands (in grey) are placed by evaporative deposition. As a result of careful tuning of the deposition parameters, palladium islands form, leaving space for ions to flow around them. Step 2**:** dry transfer emplaces a graphite crystal (in blue) with a lateral dimension of 20–50 µm. **b**, TEM images. Top, the top view of the SiN aperture after step 1; bottom, a cross-sectional view of a completed HAC device. **c**, Device and setup. Left, sketch of a finished device, with colours as in **a**. Black lines indicate typical field lines and fluidic paths. For clarity, the graphite crystal, aperture and islands are not at scale. Right, experimental setup for nanofluidic measurements. **d**, *I*–*V* characteristics (Device A, 50 mHz, 1 M KCl). The *I*–*V* curve is composed of loops self-crossing at the origin, the signature of a memristive effect. Arrows indicate the direction of the sweep. The dots show individual data points highlighting the abrupt switching and quasi-discrete conductance states. Inset, *G*–*V* curve extracted from **d**. The conductance is defined as the instantaneous current/voltage ratio, *G*(*t*) = *I*(*t*)/*V*(*t*). Here, the conductance is between 6 and 120 nS, yielding a conductance ratio of 20. Source data

To fabricate HACs (Fig. 1a and Supplementary Information Section 1), we start with silicon nitride (SiN) windows presenting a single circular aperture in their centre (step 0). A thin discontinuous layer of palladium is then evaporated (step 1). Finally, a graphite crystal (with thickness between 50 and 150 nm) is deposited above the aperture (step 2). Since step 0 and step 1 are achieved at wafer scale, this current production approach is only limited by step 2 achieved at the single-chip scale. This step is, however, a simple dry-transfer of a large pristine van der Waals crystal not requiring high positioning accuracy. The main fabrication improvement lies in eliminating the intricate etching process necessary for creating 2D channels^10,13^. With further developments, the complete nanofabrication of HACs could be realized at wafer scale. Nevertheless, the current process already enables us to produce HACs in batches of several tens of units by avoiding delicate fabrication steps at the single-device level. This scalability is required for building nanofluidic circuits, where several devices have to function simultaneously. In this study, we present results for the 32 devices referenced in Supplementary Table 1 with corresponding experiments.

Characterization using transmission electron microscopy (TEM) provides information regarding the geometry of HACs (Fig. 1b and Supplementary Figs. 2 and 3). The Pd islands have a characteristic lateral dimension of 5–10 nm and a spacing and a height of a few nanometres. Ions and water molecules experience single-digit nanometric confinement by flowing around palladium islands, converging to or diverging from the SiN aperture depending on the sign of the applied potential. HACs present a large asymmetry between their two entrances as highlighted in Fig. 1c. The inner entrance, through the membrane aperture, has a lower area *A*_in_ = π*Dh* where *h* is the island’s height and *D* is the aperture diameter. The outer entrance through the crystal edge has a larger area *A*_out_ ≈ π*Lh*, where *L* is the crystal characteristic dimension of between 20 and 50 μm. Thus, the ratio of entrance areas $\frac{L}{D}$ is on the order of several hundred, depending on the size of the top layer crystal. We expect this ratio will promote ionic accumulation when counter-ions enter the channels through the crystal edge and depletion when they enter through the aperture, according to the theoretical framework presented by ref. ^27^.

To perform nanofluidic measurements, HACs are placed into a fluidic cell separating two reservoirs filled with potassium chloride aqueous solution at pH 5.5 (Fig. 1c). Upon the application of a sinusoidal potential, HACs exhibit a clear bipolar memristive signature^27^ (the conductance increases at positive voltage and decreases at negative ones with memory retention at low voltage as shown in Fig. 1d), favourable for computing operations with programming voltage pulses. At 1 M KCl, they operate at frequencies in the 30–300 mHz range, with a conductance ratio between 9 and 60 depending on the device (Fig. 1d and Supplementary Table 1). The memory effect in HACs is therefore typically one order of magnitude stronger and two orders of magnitude faster compared to slits exhibiting similar bipolar dynamics, while presenting a larger hysteresis than micropipettes^26,27^. Our improved performance allows for complete setting and resetting HACs with 2 seconds of voltage pulses (Supplementary Fig. 6). We additionally verified that HACs can be set to intermediate levels by applying short voltage pulses, showing potential for implementing neuromorphic functions such as spike-timing-dependent plasticity (Supplementary Fig. 7). Besides exhibiting a combination of fast speed and large conductance ratio, the response of HACs also qualitatively differs from previous devices by exhibiting delayed switching^45^. When the applied voltage becomes positive, the response is initially linear until a given threshold is reached where the conductance dramatically increases (Fig. 1d). This abrupt transition from the OFF state to the ON state justifies referring to this phenomenon as ‘switching’. Such dynamics are reminiscent of solid-state electrochemical metallization memory cells^46^ and have not been reported in nanofluidics. The switching threshold (ON/OFF behaviour) enables reading the memory state without disturbing the programmed state and is beneficial for several neuromorphic computing applications, such as bio-realistic Hebbian learning or conditional logic^42,47^. These initial findings demonstrate the successful fabrication of scalable nanofluidic switches that exhibit excellent performance and operate effectively using simple monovalent salt solutions while staying within the electrochemical window of water.

**Fig. 2:**
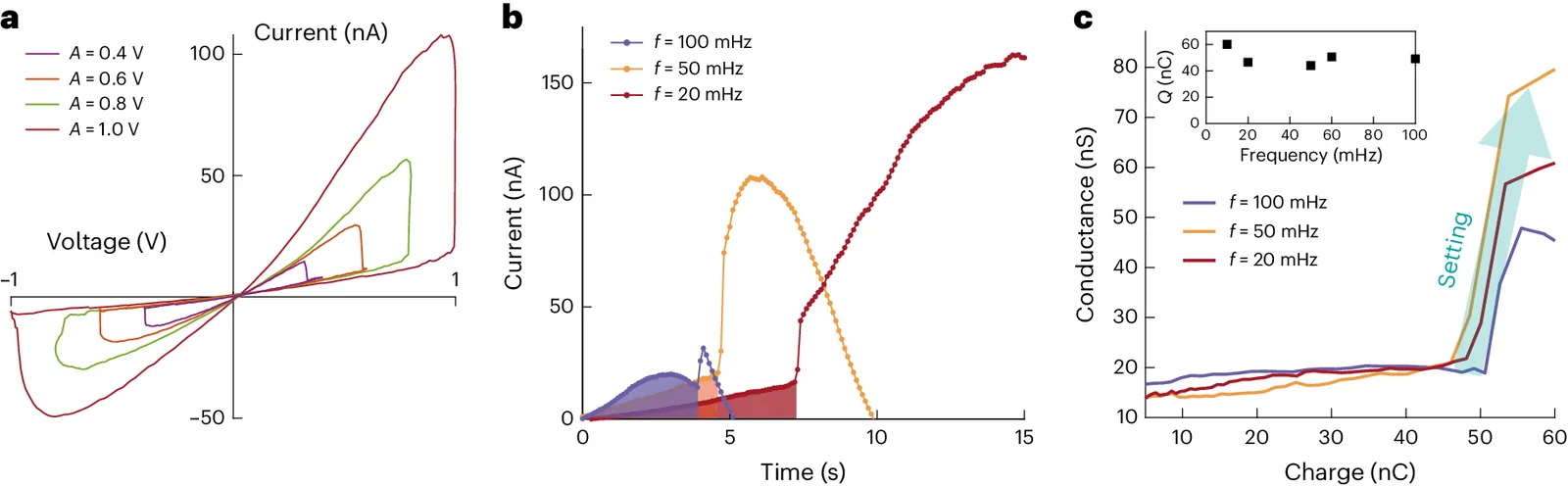
Nature of the switching threshold (Device A-1M KCl). **a**, *I*–*V* characteristics for different applied sinusoidal potential amplitudes (*A*). The applied bias frequency is 50 mHz. **b**, Device switching at different frequencies of the applied positive sinusoidal bias voltage with an amplitude of 1 V. The dots are individual data points. The shaded area represents the charge threshold $Q=\int\nolimits_{0}^{\tau }I(t){\mathrm{d}}t$, where *I(t)* is the measured current. This quantity is conserved across frequencies. **c**, Dependence of the conductance on the cumulative amount of charge, $q(t)=\int\nolimits_{0}^{t}I(t){\mathrm{d}}t$ flowing out of the device, extracted from **b**. The conductance is the instantaneous current/voltage ratio. The conductance abruptly increases when the charge threshold is reached. Here, the charge threshold is approximately equal to 50 nC. Inset, frequency dependence of the amount of charge *Q* flowing out of the device before reaching the threshold. Source data

The nature of the threshold behaviour is studied by tuning the applied sinusoidal bias *V = A* sin(ωt). The voltage at which the switching occurs depends on the bias amplitude (*A*) (Fig. 2a and Supplementary Fig. 8 with two additional devices). Consequently, switching is not triggered at a specific voltage threshold value, regardless of the chosen voltage waveform (Supplementary Fig. 9). Current traces recorded at different frequencies highlight the switching nature of ionic transport in HACs (Fig. 2b and Supplementary Fig. 11), occurring at timescale faster than 100 ms. The delay time *τ* before switching increases with decreasing frequency of the applied bias signal. Regardless of the applied frequency, the switching occurs when a given amount of charge *Q* has flowed out of the device (Fig. 2b–c). We recover such conservation of the charge threshold with three additional devices, as shown in Supplementary Fig. 11. The complete *I*–*V* cycles at various frequencies, leading to linearity at higher frequencies as expected for a memristor, are displayed in Supplementary Fig. 10 for four devices. In Supplementary Fig. 10, we also show frequency sweeps down to 1 mHz, showing a diode-like behaviour corresponding to the low-frequency limit of a charge-threshold bipolar memristor.

## Memory has a mechano–ionic origin

**Fig. 3:**
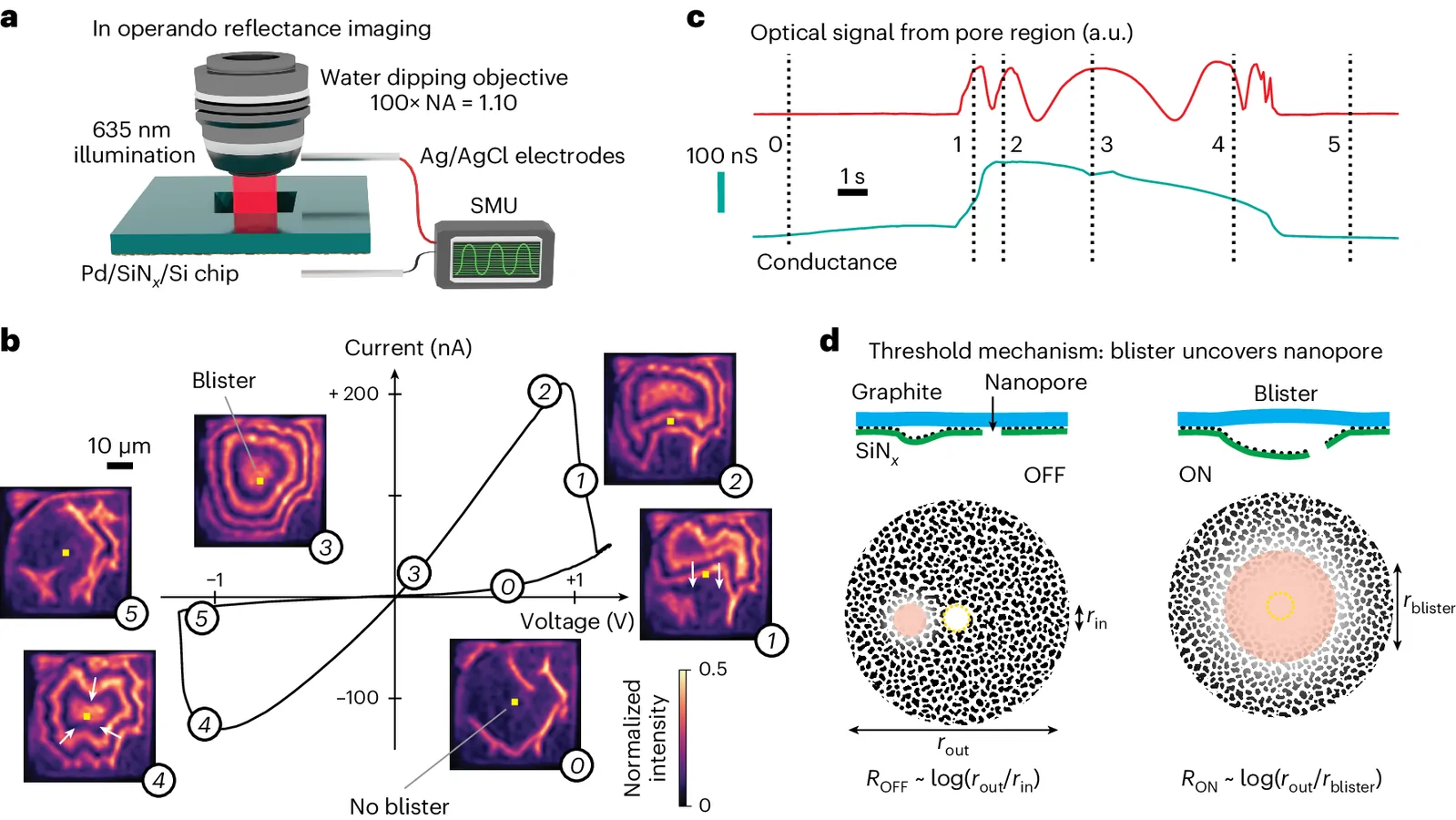
In operando microscopy reveals the memory mechanism. **a**, Setup schematic for correlative electrokinetic and optical measurements. The reflected signal from the chip backside is collected with a water-dipping objective while measuring ionic current. **b**, *I*–*V* characteristic (Device 2, 1 V, 30 mHz) with optical images of the SiN_*x*_ window at different time points marked by white dots and numbers. White arrows illustrate blister motion. **c**, Optical signal from the pore region denoted by the yellow square in the images in **b** and its correlation with the device conductance during the *I*–*V* acquisition. **d**, Threshold mechanism observed in **b** and **c**: a liquid-filled blister forms at positive bias (branch 0 to 1 in **b**) between the graphite and Pd–SiN_*x*_ walls, and the threshold occurs when the blister front crosses the nanopore (at 1 in **b**). Source data

To understand the origin of such memristive dynamics, we observe HACs in operando through wide-field light reflection imaging, as illustrated in Fig. 3a. Such direct observation of nanofluidic processes and, in particular, memory, has been identified as a major challenge to be reached for the field^28,43^. Briefly, the device chip is mounted in a custom fluidic cell similar to Fig. 1c, but with a top reservoir large enough to accommodate a water-dipping objective. The reflected light intensity from the multi-layer structure of the device is set by thin film interference, which was previously shown to enable monitoring deformations of 2D devices at the 10 nm scale^48^. See Supplementary Information Section 4.1 for details regarding in our in operando optical setup.

Our combined electrokinetic and optical results are presented in Fig. 3b. Concomitantly with the *I*–*V* curve acquisition, we observe the emergence of interference fringes at positive voltage and their disappearance at negative ones. These patterns arise from the formation of a (sub-)micrometric liquid blister between the Pd–SiN surface and the graphitic surface. Since monochromatic light is used for imaging (*λ* = 635 nm), a contrast inversion corresponds to a blister height difference of half a wavelength. The blister modulates ion conduction dramatically by uncovering the SiN aperture, which suppresses the HAC’s resistance at its bottleneck, explaining the larger conductance at positive voltage and overall bistability. As the thickness of the graphite crystal is substantially larger than the thickness of the SiN, we infer that most of the deformation arises from the SiN membrane.

For the devices in Supplementary Videos 1 and 2, the switching occurs when the blister edge moves across the SiN aperture (Fig. 3b–d). In this mechanism, the conductance state of the device correlates perfectly with the reflected intensity signal collected from the pore region (integrated in a 3 x 3 µm window around the pore), as shown in Fig. 3c. As observed in Fig. 3b and sketched in Fig. 3d, when a small off-centred blister is present, the conductance is low (OFF state, at 0), but after the threshold corresponding to the enlarged blister crossing the pore (at 1) the conductance reaches a higher value (ON state, at 2). Within the simplified radial model introduced in Supplementary Section 5, the conductance ratio can be expressed as: ${G}_{{{{\rm{ON}}}}}/{G}_{{{{\rm{OFF}}}}}=\ln ({r}_{{{{\rm{out}}}}}/{r}_{{{{\rm{in}}}}})/\ln ({r}_{{{{\rm{out}}}}}/{r}_{{{{\rm{blister}}}}})$. With *r*_in_ = 50 nm, *r*_out_ = 50 μm and *r*_blister_ = 30 μm, this formula predicts the right order of magnitude of observed conductance ratios.

In other cases, the blister forms directly above the hole and we thus attribute the switching to a sudden increase of blister volume occurring outside the SiN window (Supplementary Videos 3 and 4). This dichotomy points to the importance of optimizing HACs to control the blister motion and thereby the performance of the memristor. This will enable the building of neuromorphic nanofluidic circuits with a large number of components. All videos containing matching optical signals and *I*–*V* plots can be found in Supplementary Information.

In operando imaging shows that blisters are expanding at positive voltages and shrinking at negative voltages. We identified two plausible mechanisms to explain this phenomenology. First, the focusing of excess counter-ions can result in coulombic repulsion, overcoming the palladium–graphite adhesion near the edge of the blister. Second, as the blister morphological changes align with the expected electro-osmotic flow for negatively charged surfaces, the electro-osmotic flow could modulate the size of small-scale pre-existing blisters, by creating an effective electro-osmotic pressure that can overcome the adhesion between palladium spacers and graphite. In both cases, there is a competition between surface charge (inducing coulombic repulsion or electro-osmotic pressure) and palladium–graphite adhesion. We show in Supplementary Information Sections 5.3, 5.4 and 5.5 that electrostatic repulsion induced by charge focusing, as well as the repulsion induced by electro-osmotic pressure, could both overcome palladium–graphite adhesion. Residual strain in the SiN membrane may also play a role in the blister formation.

We verify our key assumptions with a set of control experiments. Using programming pulses, we notice HACs remain in a high conductance state after setting, similar to synaptic long-term potentiation as the blister requires a negative voltage to vanish (Supplementary Figs. 12 and 21), confirming that conductivity is directly correlated to the blister state. Pressure-driven streaming currents, as well as osmotic currents induced by the concentration gradient, show that HACs exhibit K^+^ ionic selectivity arising from a net negative surface charge (Supplementary Fig. 16). The conductance of HACs drops by two orders of magnitude between 1 M and 1 mM KCl and the memristive effect can only be observed for concentrations above 100 mM (Supplementary Fig. 14). This points to the importance of the surface charge, which—for mechanically exfoliated pristine graphite as used in this work—increases with the salt concentration to reach an absolute value above 0.1 C m^−^^2^ at 1 M (ref. ^13^). Molecular dynamics simulations also showed that the interface of pristine graphite and hexagonal boron nitride gets electrified in water^49^. While the effect can still be observed with a hexagonal boron nitride cover layer, this is not the case with mica (Supplementary Fig. 15). We also performed in operando optical measurements with mica controls, directly showing considerably reduced blister formation occurring at negative voltages, as opposed to the large deformations observed for graphite at positive voltages only. The emergence of the effect with the graphite cap at high salt concentration (equivalent to high surface charge) and the highly reduced blister dynamics with mica indicates that the memory effect requires a high charge of the top layer interface. For these reasons, as well as because their spacing is larger than twice the Debye length (0.3 nm) at 1 M, we neglect the contribution of palladium islands on the ionic transport and consider them as neutral spacers. However, they play a role on the memristive effect through their adhesion energy.

The origin of memory in HACs is thus related to reversible mechanical deformation induced by converging counter-ion fluxes, explaining the observed charge threshold for switching. These ingredients have already been used to describe out-of-the-equilibrium and nonlinear ion transport phenomena at the nanoscale^26,50^. However, the large electrostatic pressure that can build up in nanoscale systems was never reported to trigger mechanical deformations at micron scale. HACs represent the optimal geometry to favour such effects by combining large asymmetry with single-digit confinement in the presence of a highly charged interface, resulting in dramatic mechano–ionic memristive dynamics.

## Building logic circuits with interactive devices

**Fig. 4:**
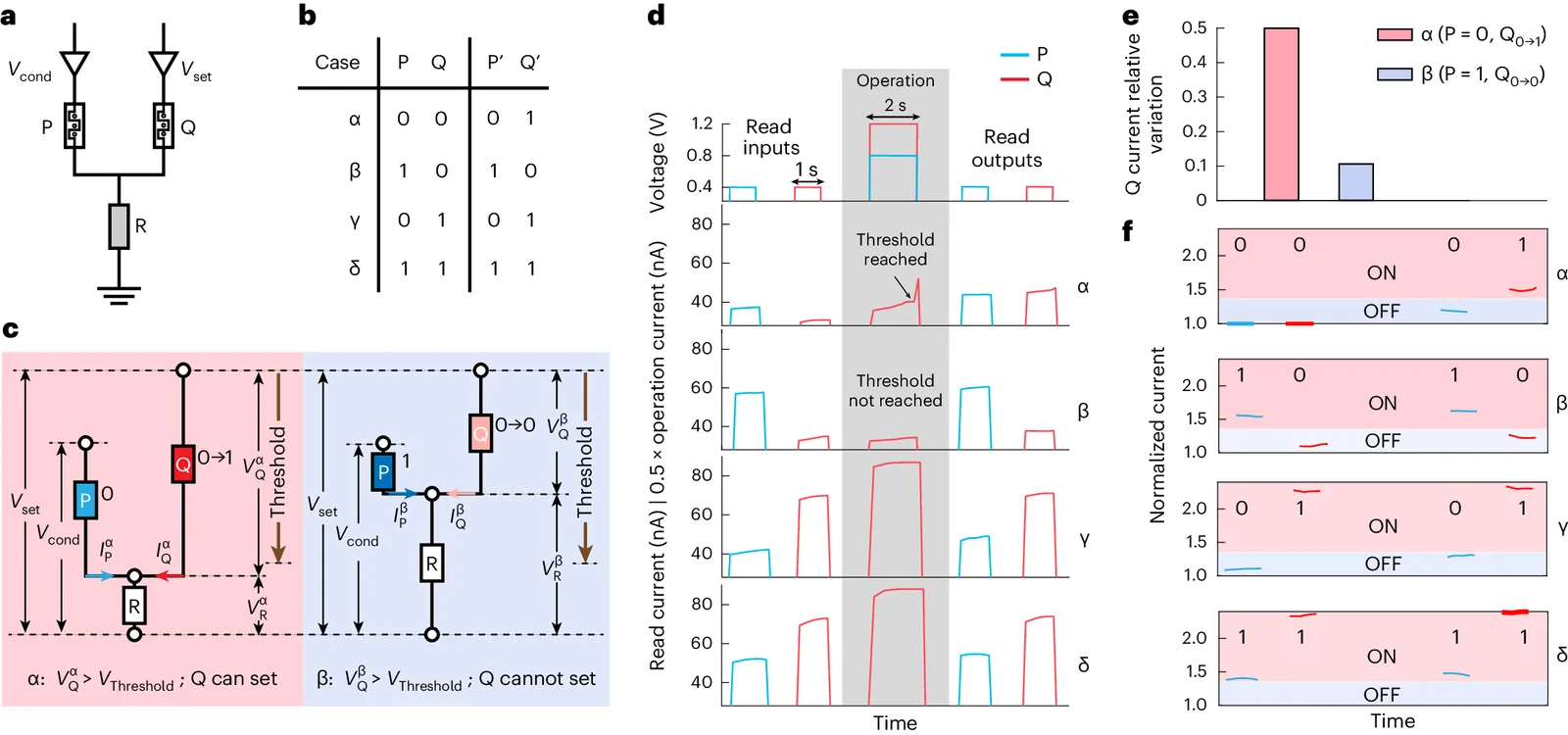
Nanofluidic logic. **a**, Circuit schematic. Two HACs are connected in parallel with a variable resistor set to 6 MΩ. The working electrodes of each device are connected to the channels of a source-measurement unit. The shared ground electrode of the two cells is connected to the resistor. **b**, IMP truth table. The first two columns (P and Q) represent input states, and the neighbouring P' and Q' columns are the corresponding outputs of the IMP gate. A Greek letter is allocated to each one of the logic cases. **c**, Illustration of the working principle of conditional switching implementing the non-trivial cases of the IMP truth table: left, the α case and right, the β case. The effective voltage *V*_Q_ applied to the Q-switch is sufficient to reach the charge threshold of the Q device within the pulse duration when P is in the 0 state (α case) and insufficient when P is in the 1 state (β case). **d**, Applied voltage and measured current for each logic case on both Q and P lines (Devices B and C, 1 M KCl). The measured pulses before the operation (with grey background) give the IMP table inputs and the ones after the operation provide the outputs. The Q-line current during operation reaches the threshold in the α case, indicated by an abrupt increase. **e**, Read current relative variations of the Q-line for the α case (rose background, P = 0) and β case, (blue background, P = 1). **f**, Read currents normalized by their respective minimum value in the α case both devices. The transition between the range corresponding to state 0 (blue background) and state 1 (rose background) occurs when the conductance of the corresponding device increases by at least 40% relative to its minimum value. Source data

Having established scalable nanofluidic device fabrication and obtained direct experimental proof regarding the underlying switching mechanism, we can utilize this knowledge to harness the potential of HACs in the realm of ionic computing. Building a logic circuit with two aqueous memristors influencing each other represents a paradigm shift for nanofluidics, where devices have until now been measured independently. We can now wire them for neuromorphic computing applications by taking advantage of the performances and reliability of HACs. This confers a new purpose to nanofluidic devices, in addition to their role as a technological platform for uncovering fundamentals of molecular transport at the smallest scales. Using two parallel HACs connected to a resistor as shown in Fig. 4a, we implement the material implication (IMP) logic gate as demonstrated by ref. ^42^ with solid-state memristors. The first HAC is called the P-switch, and its conductance state remains unchanged during the logic operation. The second HAC is the Q-switch; switching it from the OFF state (low conductance, defined as 0) to the ON state (high conductance, defined as 1) is only possible when P is in a low conductance state. Such conditional switching enables the implementation of the first two rows of the IMP truth table (Fig. 4b), which are the non-trivial cases. The two last rows are vacuous truth (or trivial cases) as Q is already switched ON at the start of the logic operation. In this demonstration of ionic computing, both nanofluidic memristors interact through the resistor to realize a conditional logic operation. The IMP gate represents a milestone for nanofluidic memristive action, as it can be used to derive any other classical logic gate commonly employed in digital computing^42^.

As HACs can be set and reset at least ten times (Supplementary Fig. 17), they are suitable for building logic circuits. We also checked the endurance of HACs under sinusoidal operation (Supplementary Figs. 18 and 23). We observe, through electrokinetic and optical measurement, perfect stability up to 17 cycles. We could also measure 300 cycles on another device, showing HACs’ robustness and setting an upper bound for nanofluidic memristors. Individual HACs can still operate when connected in series with the aforementioned resistor, even though this reduces the effective conductance ratio (Supplementary Fig. 28). To connect the switches, we use a single electrode with two chlorinated tips grounded through a variable resistor (Supplementary Fig. 27). The P and Q switches are both first placed into their targeted initial conductance state by applying a 2 s voltage pulse of high amplitude (+1 V for state 1 and −1 V for state 0). Input states of the memristors are then probed by applying read pulses to the working electrodes of each switch. The IMP gate is operated by applying voltage pulses simultaneously to each switch (Fig. 4c). In particular, a conditional voltage pulse of amplitude *V*_cond_, which is not high enough to reach the switching threshold, is applied to the working electrode of the P-switch, while a larger voltage pulse, *V*_set_ is applied to the working electrode of the Q-switch. When the P-switch is in the OFF state, the current ${I}_{{{{\rm{P}}}}}^{\upalpha }$ flowing across it is of a lower value (α case shown in the rose (left) portion of Fig. 4c). Thus, the voltage drop across the resistor ${V}_{{{{\rm{R}}}}}^{\upalpha }=R({I}_{{{{\rm{P}}}}}^{\upalpha }+{I}_{{{{\rm{Q}}}}}^{\upalpha })$ is sufficiently low, resulting in a large enough voltage drop ${V}_{{{{\rm{Q}}}}}^{\upalpha }$ across Q to reach the charge threshold required to switch Q. Conversely, when P is in the ON state (β case shown in the right panel of Fig. 4c), the ${I}_{{{{\rm{P}}}}}^{\upbeta }$ and ${V}_{{{{\rm{R}}}}}^{\upbeta }$ are larger, making the voltage drop across Q (${V}_{{{{\rm{Q}}}}}^{\upbeta }$) insufficient for switching Q. This is why a switching threshold is essential for a successful implementation of IMP logic as illustrated in Supplementary Fig. 17, where we observe in single device experiments that setting HACs is only possible with 2 s programming pulses having an amplitude high enough for the blister to reach the ON-state regime. The value of the resistor R is chosen such that the potential drop given by the low conductance state current of P is low enough to enable the pulsed setting of the Q-switch. On the other hand, the value of R needs to provide a sufficient potential drop at the high-conductance state of P to avoid the setting of Q. R is therefore chosen such that it has a value between the devices resistances in the set and conditional states of the pulsed operation, *R*_SET_ < *R* < *R*_COND_ (ref. ^42^). Finally, the output states are recorded similarly to the input states.

Figure 4d displays the experimental results obtained for the IMP gate implementation with memristive HACs. The Q-line current trace during the IMP gate operation reaches the switching threshold when P is set to 0 (α case) but not when P is set to 1 (β case). The conductance of Q increases by 50% in the α case and only 10% in the β case (Fig. 4e). We are thus able to condition the switching of a nanofluidic memristor by the conductance state of another device. We recover the IMP truth table by normalizing the read pulses of each memristor by their current minimum, while defining a posteriori an arbitrary borderline delimiting range corresponding to states 1 and 0 (Fig. 4f). Its existence serves as a proof of concept, demonstrating the potential to construct logic circuits using nanofluidic memristors as building blocks.

For robustness, we carry out another conditional switching experiment with two HACs and show switching with a HAC, as Q-switch and a variable resistor, as P-switch that emulates another HAC (Supplementary Figs. 29 and 30). We have also performed numerical simulations of an equivalent electrical circuit consisting of two charge-threshold memristive switches with typical conductance values and ratio, as in our pulse programming experiments. Qualitatively, simulation reproduces experimental results, showing that IMP logic is indeed achievable with HACs (Supplementary Fig. 31).

Logic operations with HACs are made possible by the presence of a charge switching threshold, a fast speed enabling operations with 2 s programming pulses, as well as a large conductance ratio compensating the performance loss caused by the presence of the resistor. The combination of these features is exclusive to HACs, making logic gating unattainable with previously reported nanofluidic memristors.

## Conclusions

Our HACs are scalable and compact nanofluidic memristors that can be set or reset within a few seconds, with a conductance ratio reaching sixty. In operando optical observations show that their large entrance asymmetry and single-digit confinement result in a switching behaviour related to the reversible formation of liquid blisters. This suggests several parameters that can be tuned for further optimization. In particular, choosing the membrane size and stiffness, the surface charge and adhesion properties of materials, and developing guidelines to control the blister position and dynamics will help increase performance and reliability, as well as reduce the device-to-device variation.

The strongly nonlinear dynamics of the HACs allow us to implement a Boolean operation with two interacting devices, providing the fundamental building block for future aqueous computing machines. Our HACs cannot currently match the performance of solid-state Pt/TiO2/Pt memristive switches, which operate at the microsecond timescale^42^, but further developments—such as optimizing their design and connecting them with water channels to fabricate fully liquid circuits—should lead to improvements. By taking inspiration from electronic crossbar arrays, as well as the brains of living organisms, our approach could potentially lead to the creation of nanofluidic neural networks.

## Methods

### Device fabrication

To fabricate HACs, we start with homemade SiN membranes having a single hole of approximately 100 nm width at their centre (Fig. 1a, step 0 and Supplementary Fig. 1). Using electron-beam evaporation, we then deposit a discontinuous layer of palladium that self-organizes into clusters or ‘islands’, leaving space for ions to flow around them (Fig. 1a, step 1 and Supplementary Fig. 2). Finally, we transfer a bidimensional crystal above the hole to close the system using a droplet-shaped polydimethylsiloxane stamp covered with polypropylene carbonate (Fig. 1a, step 2 and Supplementary Fig. 3). Here, we mostly used graphite but also tested the effect of using hexagonal boron nitride and mica as the top layer material. Further details are given in Supplementary Information Section 1.

### Nanofluidic measurements

Ag/AgCl electrodes are used to apply the potential and measure the resulting current. Data acquisition setup for single device measurements consists of a low noise current amplifier (FEMTO DLPCA-200, Electro Optical Components) and a digital-to-analogue converter (NI 63 series, NI). The measurements are sampled at 100 kHz with an acquisition time of 100 ms. We used KCl, CaCl_2_ and AlCl_3_ solutions with concentrations ranging from 1 mM to 1 M. For logic experiments with two channels, the working electrodes of each cell are connected to a dual channel source-measurement unit (2636B, Keithley) using a sampling rate of 10 Hz.

## Supplementary information

Supplementary Information Supplementary Figs. 1–30, Discussion and Tables 1 and 2.

Supplementary Video 1 In operando measure presented in Fig. 3 (sinusoidal).

Supplementary Video 2 In operando measurement presented in Supplementary Fig. 20 (sinusoidal).

Supplementary Video 3 In operando measurement supplementary (sinusoidal).

Supplementary Video 4 In operando measurement supplementary (sinusoidal).

Supplementary Video 5 In operando measurement presented in Supplementary Fig. 21 (pulses–retention).

Supplementary Video 6 In operando measurement presented in Supplementary Fig. 22 (mica control 1).

Supplementary Video 7 In operando measurement presented in Supplementary Fig. 22 (mica control 2).

Supplementary Data Statistical source data for Supplementary figures.

## Source data

Source Data Fig. 1 Statistical source data.

Source Data Fig. 2 Statistical source data.

Source Data Fig. 3 Statistical source data.

Source Data Fig. 4 Statistical source data.

## Data Availability

Source data are provided with this paper. Additional data related to this work are available from the corresponding authors upon request.
